# Utilization of Health Care Services and Common Disease Diagnoses among University Students: An Analysis of 35,249 Students from Thailand

**DOI:** 10.3390/ijerph18137148

**Published:** 2021-07-04

**Authors:** Suphawita Pliannuom, Kanokporn Pinyopornpanish, Chaisiri Angkurawaranon, Kanokwan Pinyopornpanish, Anawat Wisetborisut, Surinporn Likhitsathian, Wichuda Jiraporncharoen

**Affiliations:** 1 Department of Family Medicine, Faculty of Medicine, Chiang Mai University, Chiang Mai 50200, Thailand; suphawita.pliannuom@gmail.com (S.P.); chaisiri.a@cmu.ac.th (C.A.); dr.anawat@hotmail.com (A.W.); wichudaj131@gmail.com (W.J.); 2 Department of Internal Medicine, Faculty of Medicine, Chiang Mai University, Chiang Mai 50200, Thailand; kpinyopornpanish@gmail.com; 3 Department of Psychiatry, Faculty of Medicine, Chiang Mai University, Chiang Mai 50200, Thailand; surinporn.l@cmu.ac.th

**Keywords:** university students, health care service, electronic health records, epidemiology

## Abstract

The health care services for university students are important to improve student health and well-being. Analyzing the database of health conditions in the health service system will identify common health problems, which could be useful in further appropriate and specific health service planning. This study aims to investigate the utilization of health care services and common disease diagnoses among university students enrolled at Chiang Mai University during the academic year of 2018. A retrospective study was carried out using health data from the electronic health records (EHR) database of the university hospital. Ethical procedures were followed. Out of the overall 35,249 students in the academic year 2018, 17,284 students (49.03%) had visited an outpatient department (65,150 outpatient department visits), and 407 students (1.15%) had been admitted to the hospital (458 inpatient department admissions). The proportions of utilization between each field of education and training were similar across both groups. The top five categories of diagnosis, for both outpatient department visits and inpatient department admissions, differed between gender. Some of the most common diseases included trauma and injury conditions, respiratory diseases, and mental health. The conclusion of the study is that integration of a health promotion program with preventive methods, especially regarding traffic injury, transmitted diseases, mental health support, and safe environments are essential for university students. A general overview of utilization and common diseases among university students, which is still lacking in the literature, could be useful as a platform to enhance health care services for common diseases.

## 1. Introduction

With the increasing trend of enrollment in tertiary education worldwide [1], health care services for university students are important. Good services provide a supportive environment, which directly improves student health and well-being, and may also indirectly improve academic success [2]. Previous studies have shown that disease or illness in university students affected their learning [2,3,4,5], quality of life [6], and relationships [6] both during academic years and after graduation [6,7,8,9]. They may also have long-term effects on students’ health and future success [10]. For example, severe obesity during adolescence was associated with an increased risk of severe comorbidity in adults [11]. Serious physical illness in adolescence can result in decreased academic achievement and employment in adulthood [12].

The university students have been categorized into late adolescence and young adulthood, taking their individual lifecycles into account [13]. Their physical and mental health problems are different from other age groups. Common physical health problems in this age group include adolescent pregnancy, sexually transmitted disease, smoking, alcohol drinking, injury and disability due to accident, and physical violence [14,15]. Common mental health problems include depression, physical self-harm, and suicide [16,17]. However, the different social and environmental determinants of health in each country and setting may result in a varying prevalence of common health problems among university students [10].

Previous research into the utilization of health care services in university students focused mainly on the prevalence of specific diseases of interest and behaviors, including psychiatric diseases [3,4,5,9,18], substance use [19] and internet or smartphone use [20]. Previous data were also mainly derived from self-reported information. Therefore, some diseases were under-studied, and the overall data do not give a holistic picture of common diseases among university students. Accurate data pertinent to the epidemiology of health problems among large populations of university students, especially specifying common diagnosis categories, is lacking [6]. Analyzing the database of health conditions in the health service system will identify common health problems that could be useful for further appropriate and specific health service planning, for example, health promotion, disease control and prevention in identifiable high-risk groups, and early diagnosis, treatment, and rehabilitation in the case of common diseases. The present study aims to study the utilization of health care and epidemiology of health problems among university students attending Chiang Mai University (CMU), Thailand. Our research question is, what are the patterns of health care utilization and common disease diagnoses among university students?

## 2. Materials and Methods

### 2.1. Study Design

This study was a retrospective study using data from the CMU hospital’s electronic health records (EHR) during the academic year 2018 (from 31 July 2018 until 12 July 2019) [21].

### 2.2. Setting

CMU is the largest institution of higher education in the northern region of Thailand. In the Academic Year 2018 there were six education levels, including: bachelor’s degree, non-degree program, graduate certificate, master’s degree, higher graduate diploma program, and doctoral degree. There were 21 faculties and 3 colleges across a diversity of fields. The faculties included, but were not limited to, Humanities, Education, Social Sciences, Science, Engineering, Medicine, Agriculture, Economics, Architecture, Mass Communication, Political Science and Public Administration, and Law [21]. Students who attend CMU must have graduated from the high school level.

All CMU students have health insurance. This insurance is the universal health care coverage (UC) program [22]. Upon enrollment at CMU, students register CMU Hospital (Maharaj Nakorn Chiang Mai Hospital) as their primary health care provider, meaning all primary health care services, including the health promotion program, outpatient care and inpatient care, are provided through CMU Hospital. Students who were using the government civil service scheme (CSS) or social security (SS) scheme are able to register for UC under CMU Hospital when the previous benefit expires (at the age of 18 for CSS and 3 months after termination of previous employment for SS). This UC insurance with CMU Hospital is activated completely within 1 month of registration and usable until graduation [22].

CMU hospital provides services for the students at two sites. The first site is Maharaj Nakorn Chiang Mai Hospital, the teaching hospital located about 2 km from the main university campus. The second site is Phai Lom Clinic, an out-patient health center located within the main campus [22]. The students can use health services in either of these places. Phai Lom Clinic opens on weekdays from 8 a.m. to 4 p.m. At other times, they can go to the main hospital.

### 2.3. Data Sources and Contents

All student data during the academic year 2018 were derived from the registration office of CMU. The university database consists of the number of students in the academic year 2018 and some individual information about the degree and faculty of enrollment. For the purposes of this study, the fields of education and training were categorized into eight groups, in accordance with the International Standard Classification of Education (ISCED) 2013 [23]. These included: (1) Education; (2) Arts and Humanities; (3) Social Sciences, Journalism and Information; (4) Business Administration and Law; (5) Natural Science, Mathematics and Statistics; (6) Engineering, Manufacturing and Construction; (7) Agriculture and Veterinary; (8) Health Science. Additional details regarding the fields of education and training are provided in Table 1.

The clinical data of students who used health services, both outpatient department (OPD) visits and inpatient department (IPD) admissions, were derived from the EHR of the university hospital database. Students who used the health services were identified by the use of CMU student health insurance. The data collected included: gender; age; principal diagnosis in International Classification of Diseases-10 (ICD-10) diagnosis codes [24]; the visited department or admitted ward; number of visits per person; the location of receiving health service (Maharaj Nakorn Chiang Mai hospital or Phai Lom clinic); service channel (official working hours, after hours, or emergency department visit); the length of stay for IPD patients.

The diseases and diagnoses were recorded as the ICD-10 codes, a standard medical classification of disease, consisting of letters A-Z, followed by 2–4 numbers. Diagnoses from ICD-10 codes were then categorized into 22 categories of diseases by the ICD-10 chapter [24].

### 2.4. Statistical Analysis

Stata version 15.1 (Stata Corp LCC, College Station, TX, USA) software was used. Student demographic data, the clinical data of students who used health services are presented as numbers and proportions, mean and standard deviation (SD), or median and interquartile range (IQR) as appropriate. The data on the utilization of the outpatient department (OPD) and in-patient department (IPD) services were analyzed separately. Chi-square was used to evaluate the difference of health care utilization between group stratified by academic years. Common disease conditions were analyzed separately by gender and the Wald method was used for the calculation of confidence intervals.

## 3. Results

In the 2018 academic year, 35,249 students were enrolled at the university. Within one year, 17,284 students (49.03%) used the health care services provided by CMU hospital. There were 65,150 OPD visits (1.85 OPD visits per person–year) and 458 IPD admissions of 407 students (1.30 IPD admissions per 100-person–year).

### 3.1. Utilization of OPD Services

Of the 17,284 CMU students who used OPD services in CMU hospital, 63.8% were female, with an average age of 21.9 ± 3.8 years. Of the bachelor’s degree students, more than half utilized OPD services (Table 2). The proportion of students utilizing these services were higher among students in the latter stages of their degree (*p* < 0.001). Around 50% of bachelor students in their first year utilized the OPD services, while 65% of bachelor’s student in their fourth year utilized OPD services. Across different fields of education and training, the proportions of students utilizing OPD services varied between 42.6% (among students in Business Administration) and 58% (among students in Agriculture and Veterinary). Additional details regarding the utilization of OPD services by age, gender and fields of education are given in Table 2.

Table 3 presents the pattern of health care utilization among the 65,150 OPD visits. Of the students requiring the services, the mean number of visits per patient was 3.77 ± 4.53 visits (Median 2, IQR 1–4). Around 50% of patients had between 2 and 6 visits. Four percent of patients had more than 12 visits within a year (data not shown). Over 75% of the out-patient visits were at the main hospital. Nearly 80% of students accessed the health service during official working hours. One quarter of the total visits were to the campus health center. The most common diagnosis category was Z00-Z99 (factors influencing health status and contact with health services which includes services such as attention to surgical dressing and sutures, need for immunization, and issue of a medical certificate), which accounted for about 25% of all visits by both male and female students. The J00-J99 (diseases of the respiratory system) were the second most common diagnosis category, which accounted for about 15% of all visits among both male and female students. The third most common diagnosis accounted for about 8% of all visits but differed between male and female students. In males, the third most common diagnosis category was S00-T88 (injury, poisoning and certain other consequences of external causes). In women, F01-F99 (mental, behavioral, and neurodevelopmental disorders) was the third most common category.

### 3.2. Utilization of IPD Services

Four hundred and seven students accounted for 458 IPD admissions for the year. A total of 51.6% were female with an average age of 22.9 ± 4.4 years. Patients admitted to the hospital accounted for approximately 1% of the total students across different education levels and fields of education and training (Table 4). The highest proportion of students requiring admission was among those in the higher graduate diploma program students, at 3.08% (10/325 students). The majority (90.91%) were admitted once. Over half (56%) of the admissions required fewer than 3 days in hospital. Overall, 88% required fewer than 10 days of admission. The median length of stay of each admission was 3 days (IQR 2-6) (Table 5).

The top five diagnosis categories by gender are shown in Table 5. While the diagnosis of S00-T99 (injury, poisoning and certain other consequences of external causes) was the most common diagnosis in male IPD patients, accounting for 22.83% of all admissions, it is the third most common diagnosis in female IPD patients, accounting for 12.13% of all admissions. The second most common diagnosis in males was K00-K95 (diseases of the digestive system, which included such diagnosis as diseases of the appendix, intestines, gallbladder, biliary tract and pancreas, and hernia). The third most common diagnosis in males was M00-M99 (diseases of the musculoskeletal system and connective tissue). The most common diagnosis reported in female students was K00-K95 (diseases of the digestive system), accounting for 15.9% of all admissions among females, followed by C00-D40 (neoplasm), accounting for 15.1% of all admissions. Ranking fourth for women was F01-F99 (mental, behavioral, and neurodevelopmental disorders), accounting for 12.1% of all admissions.

## 4. Discussion

This study demonstrated that approximately half of the CMU students utilized health services provided by CMU. The overall utilization rate was 1.85 OPD visits per person–year. In the case of OPD, the top two services utilized for both men and women were factors influencing health status and contact with health services (Z00-Z99) and respiratory diseases (J00-J99), accounting for approximately 25% and 15% of all OPD visits respectively. About 1% of the students required hospital admission during one academic year (1.30 IPD admissions per 100-person–year). Common conditions for admissions among men and women were injury, poisoning and certain other consequences of external causes (S00-T88) and disease of the digestive system admissions (K00-K95). Relatively common causes of admission for women were mental, behavioral, and neurodevelopmental disorders (F01-F99), accounting for 12.1% of all admissions.

The present study found that the utilization rate of OPD services among CMU students was 1.85 visits per person-year, which was similar to the utilization rate of college students from the College Health Surveillance Network in the US [6]. The proportion of OPD utilization in this study, determined by hospital-based data, was very similar to a prior study carried out in Jordan that used self-reporting questionnaires rather than a database. That study noted that about 42.5% of the students reported that they had used the university health center services [25]. However, the need for hospitalization was low (less than 2%), a finding equivalent to a prior hospital-based study in Egypt [26].

Regarding the top diagnosis codes, this study shows similar results to those found in prior studies [6,27]. The top-ranking diseases leading to OPD service utilization include factors influencing health status and contact with health services, respiratory diseases, injury, infection, mental health, skin disease, and musculoskeletal diseases. In the case of IPD incidence, digestive diseases, injury and poisoning, and musculoskeletal diseases were the top-ranking diagnoses for both sexes. These results show a close correlation to those in a study from Egypt [26].

However, there are some other groups of diseases that were reported as high ranking in other studies but not in this one. For example, the study in Egypt also reported that disease of the eye and adnexa, and certain infectious and parasitic diseases, were in their top-ranking diagnoses [26]. In a study in US students, preventive-related services (e.g., contraceptive management; physical examinations for athletics, travel and work; screenings for lipid abnormality, and hypertension) had the highest percentage of diagnosis category [6]. A study from the UK reported that one of the most frequent morbidities among university students were sexual, pregnancy and childbirth consultation, and diseases of the genitourinary system [27]. These differing findings could be due to different cultures, social context, and the environmental factors in each country that might influence the individual illnesses and their access to care [28].

Some ICD-10 codes were ranked top in both OPD and IPD. These included respiratory diseases, injuries, and mental health problems. This might reflect disease progression, which may require improved health services for early detection of these diseases and to provide appropriate care in OPD settings. To improve health care services, the provision of the necessary equipment according to the top rank ICD-10 codes could improve the efficiency of treatment at the campus health centers [29,30]. The referral system to the main hospital for severe conditions with a seamless care system needs to be integrated. Population level interventions could also be useful in limiting the spread of infectious diseases, exacerbation of injuries and mental health problems [31].

Various pieces of the literature have suggested that the epidemiology of common diseases varies by gender [6]. In the present study, injuries and musculoskeletal diseases were more common among males. However, this study supported the international findings that mental health problems are relatively common among university students [32,33], ranking as one of the most common diagnoses in both OPD and IPD utilization, particularly for females. Apart from providing general health education, planning for health promotion and prevention for the specific population would provide further benefits. For males, increasing self-awareness of how to prevent possible trauma, for both traffic- and sports-related injury, should be given together with guidance on ensuring a safe environment. For mental health problems, providing appropriate health care support is essential, as it may have lifelong impact on the individual’s health [34]. Many strategies can be used, including prevention by promoting recreational activities, advocating activities to increase problem-solving skills, providing screening programs for depression and other psychiatric disorders, establishing effective systems at the faculty level to monitor and provide early treatment, and establishing mental health hotlines to provide emergency help [35].

Our findings demonstrated that the utilization of health care services is common. It is important to promote the health care system to support use by university students. The general overview of utilization and common diseases among university students, which is still lacking in the literature, could be useful as a platform to prioritize the problem and enhance health care services for common diseases. Further research should aim to study these common diseases for the future strategic planning for health promotion and disease specific treatment. Given that there is a potential difference in patterns and diseases among universities in each region of the world, we suggest that conducting similar research would provide more details on common diseases, to refine disease-specific management in each institution.

This study had several limitations. First, some CMU students may not have used the CMU student health insurance, and thus did not use health care services at the university hospital. Additionally, in relation to numbers, while some OPD attendances may be underreported, this is less likely in the case of IPD services, as these would have required out-of-pocket spending. Secondly, the ICD-10 codes of students’ diagnosis information were from the principal diagnosis alone for each visit or admission; thus, some common co-morbidities may be underreported. However, using the principal diagnosis for classification is still useful, as it usually represents the most serious disease, which requires the most resources for each visit/admission. A major strength of this study is the analysis of a large population group, including all disease diagnoses, as well as OPD and IPD utilization, to provide a more comprehensive picture.

## 5. Conclusions

The study showed that, out of 35,249 students, about half visited an outpatient department (65,150 visits in total), and about 1% were admitted (458 IPD admissions). Commonest diseases were injury conditions, respiratory diseases, and mental health problems. Treatment and preventive methods, especially in the case of traffic injury, transmitted diseases, and mental health support, are essential for university students in Thailand.

## Figures and Tables

**Table 1:** The fields of Education and Training by International Standard Classification of Education (ISCED) 2013.

Fields of Education	Faculties
Education group	Faculty of Education
2. Arts and humanities	Faculty of Humanities, Fine Arts, College of Art, Media and Technology, and International College of Digital Innovation.
3. Social sciences, journalism and information	Faculty of Social Sciences, Economics, Political Science and Public Administration, and Mass Communication.
4. Business administration and law	Faculty of Business Administration and Law.
5. Natural science, mathematics and statistics	Faculty of Science.
6. Engineering, manufacturing and construction	Faculty of Engineering, Architecture, Agro-Industry, and Biomedical Engineering Institute.
7. Agriculture and veterinary	Faculty of Agriculture and Veterinary Medicine.
8. Health science	Faculty of Medicine, Dentistry, Pharmacy, Associated Medical Sciences, Nursing, and Public Health.

**Table 2:** Socio-demographic characteristics of CMU Students utilizing OPD services (n = 17,284 students).

Characteristics	Number of Students Visiting OPD (n = 17,284)	% of Students Visiting OPD (col%)	Number of Total Students (N = 35,249)	% of Total Students (row%)
Sex				
- Male	6262	36.23	NA *	NA *
- Female	11,022	63.77		
Age (Year)				
- <21	6484	37.51	NA *	NA *
- 21–35	10,526	60.90		
- 36–50	259	1.50		
- 50–60	14	0.08		
- >60	1	0.01		
- Mean (SD)	21.93 (3.77)		NA *	NA *
- Median (IQR)	21 (20,23)			
Student level				
1. Bachelor’s degree (Year of study)				
**- 1 year**	3740	21.64	7372	50.73
**- 2 years**	3233	18.71	6395	50.56
**- 3 years**	3359	19.43	6342	52.96
**- 4 years**	3308	19.14	6407	51.63
**- 5 years**	1073	6.21	1639	65.47
**- 6 years**	622	3.60	816	76.23
**- >6 years**	7	0.04	216	3.24
2. Non-degree program	39	0.23	88	44.32
3. Graduate Certificate	4	0.02	29	13.79
4. Master’s degree	1,315	7.61	4,316	30.47
5. Higher Graduate Diploma Program	285	1.65	325	87.69
6. Doctoral degree	299	1.73	1304	22.93
The Fields of education and training (ISCED 2013)				
- Education	1017	5.88	1971	51.60
- Arts and humanities	2519	14.57	5224	48.22
- Social sciences, journalism and information	2162	12.51	4744	45.57
- Business administration and law	1586	9.18	3739	42.42
- Natural science, mathematics and statistics	1614	9.34	3048	52.95
- Engineering, manufacturing and construction	2854	16.51	6165	46.29
- Agriculture and veterinary	1284	7.43	2214	57.99
- Health science	4248	24.58	8144	52.16

* NA means the data is not available in the database.

**Table 3:** The characteristics of utilization of OPD services (17,284 students with 65,150 visits).

Characteristic	Utilization of OPD Services		
	n	%	
Visits per patient (n = 17,284 students)			
- 1 visit	5948	34.31	
- > 1 visit	11,336	65.59	
- Mean (SD)	3.77 (4.53)		
- Median (IQR)	2 (1,4)		
Setting (N = 65,150 visits)			
- Phai Lom clinic	14,498	22.25	
- Maharaj Nakorn Chiang Mai hospital	50,652	77.75	
Time of attendance (N = 65,150 visits)			
- Official working hours	50,870	78.08	
- After hours	10,215	15.68	
- Emergency room (24 h)	4065	6.24	
The top 5 ICD coding categorized by gender (N = 58,180 visits) *			
Male (n = 21,769 visits)	n	%	95%CI
1. Z00-Z99: Factors influencing health status and contact with health services	5508	25.30	24.73–25.89
2. J00-J99: Diseases of the respiratory system	3416	15.69	15.21–16.18
3. S00-T88: Injury, poisoning and certain other consequences of external causes	1880	8.64	8.27–9.02
4. A00-B99: Certain infectious and parasitic diseases	1795	8.25	7.88–8.62
5. M00-M99: Diseases of the musculoskeletal system and connective tissue	1463	6.72	6.39–7.06
Female (*n* = Total 36,411 visits)	*n*	%	95%CI
1. Z00-Z99: Factors influencing health status and contact with health services	8908	24.47	24.02–24.91
2. J00-J99: Diseases of the respiratory system	5145	14.13	13.77–14.49
3. F01-F99: Mental, Behavioral, and Neurodevelopmental disorders	2980	8.18	7.90–8.47
4. S00-T88: Injury, poisoning and certain other consequences of external causes	2537	6.97	6.71–7.23
5. L00-L99: Diseases of the skin and subcutaneous tissue	2112	5.80	5.56–6.05

* The ICD-10 coding was missing in 6970 visits.

**Table 4:** Socio-demographic characteristics of CMU Students utilizing IPD service (n = 407 students).

Characteristics	Number of Students Admitted (n = 407)	% of Admitted Students	Number of Total Students (N = 35,249)	% of Total Students
Sex				
- Male	197	48.40	NA *	NA *
- Female	210	51.60		
Age (Year)				
- <21	99	24.32	NA *	NA *
- 21–35	295	72.48		
- 36–50	13	3.19		
- >50	0	0.00		
- Mean (SD)	22.95 (4.43)		NA *	NA *
- Median (IQR)	22 (21,23)			
Student level				
1. Bachelor’s degree (Year of study)				
**- 1 year**	57	14.00	7372	0.77
**- 2 years**	82	20.15	6395	1.28
**- 3 years**	68	16.71	6342	1.07
**- 4 years**	87	21.38	6407	1.35
**- 5 years**	25	6.14	1639	1.53
**- 6 years**	18	4.42	816	2.21
**- >6 years**	0	0.00	216	0.00
2. Non-degree program	1	0.25	88	1.14
3. Graduate Certificate	0	0.00	29	0.00
4. Master’s degree	53	13.02	4316	1.23
5. Higher Graduate Diploma Program	10	2.46	325	3.08
6. Doctoral degree	6	1.47	1304	0.46
The fields of education and training (ISCED 2013)				
- Education	31	7.62	1971	1.57
- Arts and humanities	71	17.44	5224	1.36
- Social sciences, journalism and information	46	11.30	4744	0.97
- Business administration and law	34	8.35	3739	0.91
- Natural science, mathematics and statistics	52	12.78	3048	1.71
- Engineering, manufacturing and construction	73	17.94	6165	1.18
- Agriculture and veterinary	27	6.63	2214	1.22
- Health science	73	17.94	8144	0.90

* NA means the data is not available in the database.

**Table 5:** The characteristics of utilization of IPD services (407 students with 458 admissions).

Characteristic	Utilization of IPD Services		
	n		%
Number of admissions per patient (407 students)			
- 1 time	370		90.91
- >1 time	37		9.09
- Mean (SD)	1.13 (0.49)		
- Median (IQR)	1 (1,1)		
Days of stay (458 admissions)			
- 3 days or less	261		56.99
- 4–6 days	100		21.83
- 7–9 days	39		8.52
- 10 days or more	59		12.66
- Mean (SD)	5.17 (6.27)		
- Median (IQR)	3 (2,6)		
The top 5 ICD coding categorized by gender (458 admissions)			
Male (219 admissions)	n	%	95%CI
1. S00-T88: Injury, poisoning and certain other consequences of external causes	50	22.83	17.45–28.97
2. K00-K95: Diseases of the digestive system	35	15.98	11.39–21.52
3. M00-M99: Diseases of the musculoskeletal system and connective tissue	27	12.33	8.28–17.43
4. G00-G99: Diseases of the nervous system	21	9.59	6.03–14.28
5. J00-J99: Diseases of the respiratory system	16	7.31	4.23–11.59
Female (239 admissions)	n	%	95%CI
1. K00-K95: Diseases of the digestive system	38	15.90	11.50–21.16
2. C00-D40: Neoplasms	36	15.06	10.78–20.24
3. S00-T88: Injury, poisoning and certain other consequences of external causes	29	12.13	8.28–16.96
4. F01-F99: Mental, Behavioral and Neurodevelopmental disorders	29	12.13	8.28–16.96
5. M00-M99: Diseases of the musculoskeletal system and connective tissue	19	7.95	4.85–12.14

## Data Availability

Not applicable.

## References

[1] Statistics UIf (2020). School Enrollment, Tertiary (% Gross). https://data.worldbank.org/indicator/SE.TER.ENRR.

[2] El Ansari W., Stock C. (2010). Is the health and wellbeing of university students associated with their academic performance? Cross sectional findings from the United Kingdom. Int. J. Environ. Res. Public Health.

[3] Auerbach R.P., Alonso J., Axinn W.G., Cuijpers P., Ebert D.D., Green J.G., Hwang I., Kessler R.C., Liu H., Mortier P. (2016). Mental disorders among college students in the World Health Organization World Mental Health Surveys. Psychol. Med..

[4] Bruffaerts R., Mortier P., Kiekens G., Auerbach R.P., Cuijpers P., Demyttenaere K., Green J.G., Nock M.K., Kessler R.C. (2018). Mental health problems in college freshmen: Prevalence and academic functioning. J. Affect. Disord..

[5] Ketchen Lipson S., Gaddis S.M., Heinze J., Beck K., Eisenberg D. (2015). Variations in student mental health and treatment utilization across US colleges and universities. J. Am. Coll. Health.

[6] Turner J.C., Keller A. (2015). College health surveillance network: Epidemiology and health care utilization of college students at US 4-year universities. J. Am. Coll. Health.

[7] Vaughn A., Drake R.R., Haydock S. (2016). College student mental health and quality of workplace relationships. J. Am. Coll. Health.

[8] Garcia-Williams A.G., Moffitt L., Kaslow N.J. (2014). Mental health and suicidal behavior among graduate students. Acad. Psychiatry.

[9] Volpe U., Ventriglio A., Bellomo A., Kadhum M., Lewis T., Molodynski A., Sampogna G., Fiorillo A. (2019). Mental health and wellbeing among Italian medical students: A descriptive study. Int. Rev. Psychiatry.

[10] Oswalt S.B., Lederer A.M., Schrader L.T. (2015). Institutional characteristics and the connection to college student health. Am. J. Health Behav..

[11] Inge T.H., King W., Jenkins T.M., Courcoulas A.P., Mitsnefes M., Flum D.R., Wolfe B.M., Pomp A., Dakin G.F., Khandelwal S. (2013). The effect of obesity in adolescence on adult health status. Pediatrics.

[12] Hale D.R., Viner R.M. (2018). How adolescent health influences education and employment: Investigating longitudinal associations and mechanisms. J. Epidemiol. Community Health.

[13] Armstrong T. (2006). The Human Odyssey: Navigating the Twelve Stages of Life.

[14] Office NS (2017). The Results of the Survey on the Fertility Rate of Adolescents Aged 15–19 Years: Survey of the Situation of Children and Women in Thailand 2015–2016. https://www.hfocus.org/content/2017/11/14919.

[15] University IfPaSRM (2019). Thai Health Report 2019: Social Media, Double-Sided Media, Well-Being of Thai People in Social Media.

[16] Department of Mental Health MoPH (2018). Annual Report of the Department of Mental Health, Year 2018.

[17] Mongkol A. (2011). Report of the Number of Suicides in Thailand. https://www.dmh.go.th/report/suicide/age.asp.

[18] Torres C., Otero P., Bustamante B., Blanco V., Díaz O., Vázquez F.L. (2017). Mental health problems and related factors in Ecuadorian college students. Int. J. Environ. Res. Public Health.

[19] Ayalew M., Tafere M., Asmare Y. (2018). Prevalence, trends, and consequences of substance use among university students: Im-plication for intervention. Int. Q. Community Health Educ..

[20] Carbonell X., Chamarro A., Oberst U., Rodrigo B., Prades M. (2018). Problematic use of the internet and smartphones in university students: 2006–2017. Int. J. Environ. Res. Public Health.

[21] Office CMR (2017). Chiang Mai: Registration Office. http://www1.reg.cmu.ac.th.

[22] (2019). Health Services for Students of Chiang Mai University 2019. https://cmu.ac.th/content/FirstYear/MentalHealthServices2564.pdf.

[23] UNESCO Institute of Statistics (2013). International Standard Classification of Education (ISCED). http://uis.unesco.org/en/topic/international-standard-classification-education-isced.

[24] (2019). International Statistical Classification of Diseases and Related Health Problems 10th Revision. https://icd.who.int/browse10/2019/en.

[25] Alkhawaldeh A. (2017). Utilisation of university health care centre services among university students. Int. J. Health Sci. Res..

[26] El-Gilany A.-H., El-Masry R., Badawy K. (2014). Students’ utilization of health services: A hospital-based study in Mansoura Uni-versity, Egypt. Eur. J. Gen. Med..

[27] Finlay S.E. (1976). Physical diseases in university students. Br. Med. J..

[28] Tervalon M. (2003). Components of culture in health for medical students’ education. Acad. Med. J. Assoc. Am. Med. Coll..

[29] Srakshetrin A., Thongphet P., Kochanam S., Vatchalavivat A., Sukgree J., Ratananugool N., Choptong S. (2004). Factors Affecting Health Promotion Behaviors and Health Service Use Behaviors of People Under the Universal Health Coverage. https://kb.hsri.or.th/dspace/handle/11228/1446?fbclid=IwAR0mCwJL-n0qFY_Q6R1ew2frY4_Q52EuRnlBH4aOLPJK0M3S4cLJQcsMdHA.

[30] Jangwechchai B. (2016). Service Quality Affecting the Satisfaction of BTS SkyTrain Passengers in Bangkok. Master’s Thesis.

[31] Sawyer M.G., Borojevic N., Lynch J. (2011). Evaluating population-level interventions for young people’s mental health: Challenges and opportunities. Early Interv. Psychiatry.

[32] Altemus M., Sarvaiya N., Epperson C.N. (2014). Sex differences in anxiety and depression clinical perspectives. Front. Neuroendocr..

[33] Takahashi A. (2021). Toward understanding the sex differences in the biological mechanism of social stress in mouse models. Front. Psychiatry.

[34] Phillips S.P., Reipas K., Zelek B. (2019). Stresses, strengths and resilience in adolescents: A qualitative study. J. Prim. Prev..

[35] World Health Organization (2004). Prevention of Mental Disorders: Effective Interventions and Policy Options: Summary Report/A Report of the World Health Organization Dept. of Mental Health and Substance Abuse; in Collaboration with the Prevention Research Centre of the Universities of Nijmegen and Maastricht.

